# Language Use in Conversational Agent–Based Health Communication: Systematic Review

**DOI:** 10.2196/37403

**Published:** 2022-07-08

**Authors:** Yi Shan, Meng Ji, Wenxiu Xie, Xiaobo Qian, Rongying Li, Xiaomin Zhang, Tianyong Hao

**Affiliations:** 1 School of Foreign Studies Nantong University Nantong China; 2 School of Languages and Cultures University of Sydney Sydney Australia; 3 Department of Computer Science City University of Hong Kong Hong Kong China; 4 School of Computer Science South China Normal University Guangzhou China; 5 School of Artificial Intelligence South China Normal University Guangzhou China; 6 Department of Linguistics Macquarie University Sydney Australia

**Keywords:** systematic review, health communication, language use, conversational agent

## Abstract

**Background:**

Given the growing significance of conversational agents (CAs), researchers have conducted a plethora of relevant studies on various technology- and usability-oriented issues. However, few investigations focus on language use in CA-based health communication to examine its influence on the user perception of CAs and their role in delivering health care services.

**Objective:**

This review aims to present the language use of CAs in health care to identify the achievements made and breakthroughs to be realized to inform researchers and more specifically CA designers.

**Methods:**

This review was conducted by following the protocols of the PRISMA (Preferred Reporting Items for Systematic Reviews and Meta-Analyses) 2020 statement. We first designed the search strategy according to the research aim and then performed the keyword searches in PubMed and ProQuest databases for retrieving relevant publications (n=179). Subsequently, 3 researchers screened and reviewed the publications independently to select studies meeting the predefined selection criteria. Finally, we synthesized and analyzed the eligible articles (N=11) through thematic synthesis.

**Results:**

Among the 11 included publications, 6 deal exclusively with the language use of the CAs studied, and the remaining 5 are only partly related to this topic. The language use of the CAs in these studies can be roughly classified into six themes: (1) personal pronouns, (2) responses to health and lifestyle prompts, (3) strategic wording and rich linguistic resources, (4) a 3-staged conversation framework, (5) human-like well-manipulated conversations, and (6) symbols and images coupled with phrases. These derived themes effectively engaged users in health communication. Meanwhile, we identified substantial room for improvement based on the inconsistent responses of some CAs and their inability to present large volumes of information on safety-critical health and lifestyle prompts.

**Conclusions:**

This is the first systematic review of language use in CA-based health communication. The results and limitations identified in the 11 included papers can give fresh insights into the design and development, popularization, and research of CA applications. This review can provide practical implications for incorporating positive language use into the design of health CAs and improving their effective language output in health communication. In this way, upgraded CAs will be more capable of handling various health problems particularly in the context of nationwide and even worldwide public health crises.

## Introduction

### Background

Conversational agents (CAs) are intelligent computer programs empowered with natural language processing techniques that engage users in human-like conversations to provide an effective and a smart communication platform in a simulated environment, including text-based chatbots, voice-activated assistants, and embodied CAs [1,2]. They are designed to obtain specific information from users that is necessary to perform particular tasks and respond in a manner that is optimal to achieve these goals. Due to their ability to transform the health care system and enable individuals to comanage their health care effectively, CAs are increasingly used to deliver health care services [3]. The most popular health CAs include ELIZA [4], Casper [5], MedChat [5], PARRY [6], Watson Health [7], Endurance [7], OneRemission [8], Youper [9], Florence [10], Your.Md [11], AdaHealth [12], Sensely [13], and Buoy Health [14], among many others. CAs are being tested and adopted to provide and collect health-related information and provide treatment and counseling services [15]. In some cases, they are used to enhance the accessibility, efficiency, and personalization of service delivery and ensure relatively equal delivery of health care services worldwide through bridging the gaps between developing and developed countries [15,16].

Given the growing significance of CAs, researchers have conducted a plethora of relevant studies, varying from their suitability as health care partners to their designs including physical appearance, gender, and speech. [17-20]. These studies aimed to improve “humanness heuristics,” affective states in users, and user perceptions of the CA personalities by tailoring CAs to the cultures and demographics of the users to continuously promote user engagement, adherence, and adoption [21-24].

Language plays a crucial role in improving user engagement because perceived impersonal closeness, intention to use, user satisfaction, establishment of trust, and user self-disclosure or self-concealment are closely associated with the task- and social-based interactivity, interaction, politeness, and information quality provided by CAs [2,19,21,25-32]. However, few studies focused on language use in CA-based health communication to examine its influence on the perceived usability of CAs and the perceived roles of CAs in delivering health care services [16]. Language considerably influences the joint construction of meaning between interlocutors and rapport establishment [23,33,34]. This is particularly true for human-machine communication. For example, when addressing users by their first names, CAs are perceived to display varying degrees of politeness and thoughtfulness determined by cultural limits and preferences [24]. It follows that intensive and extensive investigations into language use by CAs in different linguistic settings are crucial to scale up health care interventions delivered by CAs worldwide [16,35].

### Language Use and Its Significance in CA Communication

The language use of an information source is likely to be crucial among various factors affecting the information seekers’ judgments on the credibility and trustworthiness of the information providers [36-39]. In this review, language use, characterized by various linguistic aspects, is defined as varied verbal strategies and compliance-gaining techniques [40] that the CAs under scrutiny adopted to deliver health interventions. These strategies and techniques may involve various ways of wording, including an everyday style (eg, “heart attack”) versus a technical style (eg, “myocardial infarction”), a tentative style (eg, “presumably similar”) versus a nontentative style (eg, “similar”), a neutral style (eg, “methodological mistakes”) versus an aggressive style (eg, “really dumb methodological mistakes”), an emotional style versus a nonemotional style, and an enthusiastic style versus a nonenthusiastic style. [36,41-44]. They may also include the use of personal references (eg, first-person and second-person pronouns), personal testimonials, specific conversational frameworks or prompts, and other verbal means of communication [45-47]. In short, the language use of the CAs under discussion in this review refers to their characteristic linguistic performances in health communication.

Language Expectancy Theory [48] and Communication Accommodation Theory [49] assert that acquiring knowledge when seeking web-based health information is determined not only by the information content but also by who is communicating the information and the manner and context of communication. Information seekers evaluate information providers positively if the latter’s language use is in tune with their cultural values and situational norms and if they use language more favorably than expected in a situation [50]. The language use of information providers is regarded as a prominent clue to evaluate the characteristics of the providers, especially in web-based communication [51,52]. The information provider’s language use is a cue for determining whether people perceive the information to be credible and whether the information provider is trustworthy [37,46].

### Objective

The current review aimed to summarize the language use of CAs in health care to identify the achievements made and the breakthroughs to be made to inform researchers and more particularly CA designers and developers. This can help realize the high potential of CAs for improving individual well-being.

## Methods

### Study Design

The primary objective of the current review was to identify the language use of CAs in health care. This review was performed by following the protocols of the PRISMA (Preferred Reporting Items for Systematic Reviews and Meta-Analyses) 2020 statement [53]. We first designed a search strategy according to the research aim and then performed keyword searches in PubMed and ProQuest databases for retrieving relevant publications. Then, 3 researchers screened and reviewed the publications independently to select studies meeting the predefined selection criteria. Finally, we synthesized and analyzed the eligible articles.

### Search Strategy and Study Selection Criteria

This review focused on two aspects of the previous studies: CA applications in health care and language use. To retrieve a high number of relevant studies, we decided on using the keywords relating to language use for literature search, including “expression,” “language,” “language style,” “language feature,” “language characteristic,” “language pattern,” “linguistic style,” “linguistic feature,” and “linguistic characteristic.” Based on these keywords and those concerned with CA applications in health care, we developed the following search strategy to identify studies wholly or partly investigating the language use of CAs: ((expression [Title/Abstract]) OR (language [Title/Abstract]) OR (language style [Title/Abstract]) OR (language feature [Title/Abstract]) OR (language characteristic [Title/Abstract]) OR (language pattern [Title/Abstract]) OR (linguistic style[Title/Abstract]) OR (linguistic feature [Title/Abstract]) OR (linguistic characteristic [Title/Abstract])) AND ((health* chatbot [Title/Abstract]) OR (health* conversational agent [Title/Abstract])). Drawing on this search strategy, we conducted keyword searches in 2 databases (PubMed and ProQuest) to retrieve published papers without restrictions regarding the year of publication on February 11, 2022.

We included both peer-reviewed and non–peer-reviewed journal publications because the aim of this review was to provide a comprehensive overview of the language use of CAs in health care and its corresponding implications for improvement in language use in CA communication to inform future research and CA designers. Textbox 1 shows the inclusion and exclusion criteria.

**Inclusion and exclusion criteria of the study.**

**Inclusion criteria**

- Articles wholly or partly examining the language use of conversational agents (CAs) in health care were included.
- Articles on CAs that are equipped with languages other than English were included.

**Exclusion criteria**

- Publications that are not journal articles (eg, reports, editorials, dissertations, and news) were excluded.
- Articles that were not written in English were excluded.
- Articles that focus on the development of CAs and do not cover any design or setting of system-human linguistic interactions were excluded.
- Studies that examine the application of CAs in other fields than health care were excluded.

### Article Selection and Data Extraction

We used Microsoft Excel (Microsoft Corporation) to manage the collected articles by listing the titles, abstracts, and article types for screening. First, 2 researchers (MJ and YS) screened the titles and abstracts of the candidate articles independently, filtering those articles that did not conform to the selection criteria. If the eligibility of some studies was unclear, we included them for further full-text review. Then, 2 researchers (YS and WX) reviewed the full texts of the remaining articles independently. Any disagreements were resolved through discussion and consultation with the third researcher (MJ).

To analyze and synthesize the language use of the health care CAs, the following information was extracted from eligible studies by YS: first author, year of publication, health care application, target population, study design, major findings, and limitations. Then, MJ reviewed and cross-checked the extracted data. Any discrepancies were resolved through a discussion with the entire research team.

### Data Analysis and Synthesis

A meta-analysis was not feasible due to the expected variety of health care applications, target populations, study designs, results, and limitations. Therefore, we conducted thematic synthesis to summarize the data extracted from the included articles following 3 steps, namely “line-by-line” coding of the text, development of “descriptive themes,” and generation of “analytical themes” [54]. YS first coded each line of the extracted text according to its meaning, then developed descriptive themes, and finally generated analytical themes using the derived descriptive themes [55]. MJ validated each assigned code, each derived descriptive theme, and each developed analytical theme independently. All the authors discussed and finalized the results of the thematic synthesis.

## Results

### Search Results

**Figure 1:**
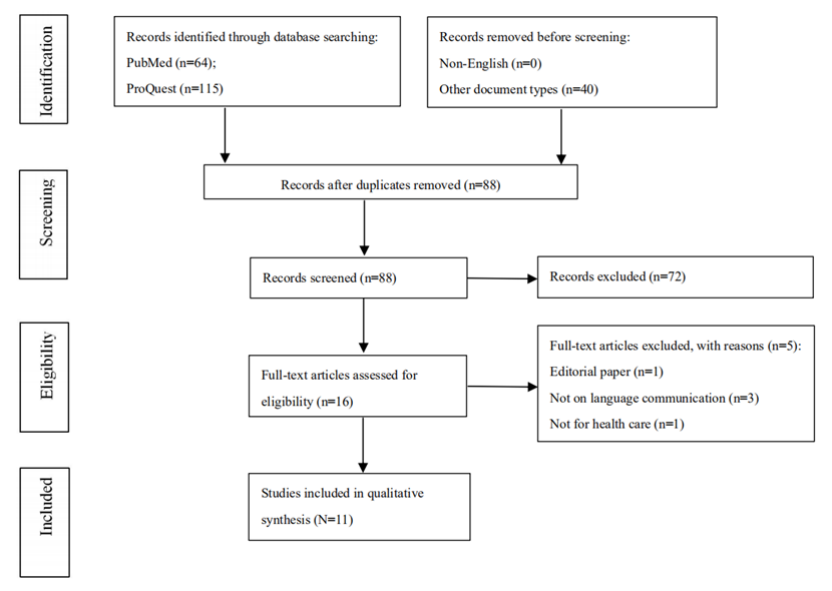
PRISMA (Preferred Reporting Items for Systematic Reviews and Meta-Analyses) flowchart of the selection of eligible studies.

Using the search strategy, we identified 179 publications in the PubMed and ProQuest databases. From these retrieved publications, 40 were eliminated because they were not journal articles but were other types of publications (eg, commentaries, letters, news, and editorials); 51 were eliminated for being duplicates, and 72 for not meeting the selection criteria. After the full-text review, another 5 studies were excluded; 3 were not related to language communication, 1 was not about health care, and 1 was an editorial. As a result, 11 studies met the inclusion criteria and were eligible to be considered in this systematic review. Figure 1 shows the screening and selection process.

### Characteristics of Included Studies

Table 1 summarizes the information extracted from the 11 papers selected for synthesis and analysis. The major findings reported in the original studies that are directly related to the aim of the current review are included. We have included the limitations reported in the original studies and those based on our perspectives, if any. Based on this table, we present the qualitative synthesis and analysis in the Discussion section.

**Table 1:** Information extracted from the 11 selected studies.

Reference, first author, and year of publication	Health care application	Target population	Study design	Major findings	Limitations
[56]; Ollier; 2022	A public health CA^a^ prototype	People in French and German lingua cultures	An internet-based experiment	The CA’s choice of formal and informal forms of the second-person pronoun “You”— *Tu/Vous* (T/V) distinction—affected the evaluations of users of different ages, genders, and cultures to varying degrees.	Given that the study involved a complicated 4-way interaction between T/V distinction, language and culture, age, and gender, the sample size is not sufficiently large to ensure more generalizable findings. Therefore, the implications for CA designers are affected.
[57]; Kocaballi; 2020	Commonly available, general-purpose CAs on smartphones and smart speakers	Unspecified	Following a piloted script to present health- and lifestyle-related prompts to 8 CAs	The ratio of the CAs’ appropriate responses decreased when safety-critical prompts were rephrased or when the agent used a voice-only interface. The appropriate responses included mostly directive content and empathy statements for the safety-critical prompts and a mix of informative and directive content for the lifestyle prompts.	Some response structures were derived from the patterns observed in the responses to a reasonably limited set of studied prompts, possibly not capturing additional or different structural elements of the CAs’ responses based on a larger set of prompts. CAs failed to provide a larger amount of precoded information on some safety-critical prompts.
[58]; Boustani; 2021	An expressive, speech-enabled digital health agent to deliver an internet-based brief behavioral health intervention for alcohol use	51 alcohol users in the United States	Description of the CA design, acceptability, feasibility, and utility	The CA used a model of empathetic verbal and nonverbal behaviors to engage users, who had overwhelmingly positive experiences with the digital health agent, including engagement with the technology, acceptance, perceived utility, and intent to use the technology.	It is unclear whether the model of empathetic verbal and nonverbal behaviors the CA used to engage young and middle-aged adults successfully can equally engage the elderly or children in America and users in other countries, especially considering different cultural factors that may influence the perception of language.
[59]; Miner; 2016	68 phones from 7 producers	Investigators	A pilot study followed by a cross-sectional study	Some CAs replied to users’ concerns with respectful language and referred them to helplines, emergency services, and nearby medical facilities, but some failed to do so.	Investigators used standardized phrases for health and interpersonal violence concerns, but people asking for help on their personal smartphones may use different phrases, which may influence the CAs’ responses. The study only tested a limited number of CAs available in the United States and evaluated their responses to a limited number of health concerns, which may affect the generalizability of the findings.
[60]; Grové; 2020	A mental health and well-being chatbot named Ash	Young people aged 15-17 years and living in Australia	Interviews and a survey	The chatbot failed to identify and understand critical words and generate responses appropriate to critical words. Exemplary dialogs show the chatbot’s respectful, empathetic, supportive, and encouraging language style.	The imbalanced numbers of male and female participants who interacted with the chatbot may influence the responses of the chatbot.
[61]; Ireland; 2015	Chatbots for people with Parkinson disease	People with Parkinson disease	A description of chatbots for people with Parkinson disease	The chatbots can engage with patients in random, human-like conversations.	The study failed to cite chatbot-patient conversations to illustrate the randomness and human-likeness of the conversations.
[62]; Frick; 2021	CAs	German participants	An internet-based questionnaire and a comparative study	Only an exemplary anamnesis with a CA shows the CA’s polite, respectful, and encouraging language style.	The study failed to discuss the role of the CA’s language style in soliciting disclosure of medical information from patients.
[63]; Cooper; 2018	A chatbot named Alex	Children on the autism spectrum	A description of a chatbot	The chatbot is able to engage with the user on a variety of topics using symbols and images.	The study aimed to describe a new chatbot and did not provide real-time exemplary conversations between the chatbot and real patients, making it difficult for us to understand the role of its language style in engaging patients.
[64]; Almushrraf; 2020	A motivational interviewing–based chatbot	Adult cigarette smokers	A single-arm prospective iterative design study	Due to the running head start technique that the chatbot used when engaging in conversations, 34.7% (42/121) of participants enjoyed the interaction with the chatbot. The chatbot finished the conversation after receiving the response to the exception case questions.	The running head start technique might not be appropriate or helpful for those who were already exhibiting change behavior. The lack of follow-on to the exception case questions or elsewhere in the conversation can frustrate subjects and possibly lead to negative unintended effects.
[65]; Ireland; 2016	An artificial CA named Harlie	People with neurological conditions such as Parkinson disease and dementia	A description of a chatbot	The chatbot is able to converse with the user on a variety of topics. It can engage patients with a random, human-like, well-manipulated conversation style to gain information about challenges patients encounter and play an educational and supportive role.	The study focused on the chatbot’s role in performing different tasks without attaching importance to the function of its language style in engaging the patients.
[66]; Ireland; 2021	A trainee chatbot named Edna	5 genetic counselors and adults who had whole exome sequencing conducted for diagnosis of a genetic condition, either for themselves or their child	A description of a chatbot	The chatbot can engage users with a polite, respectful, and an encouraging language style. The chatbot can educate users through explaining genetic conditions and terminologies precoded into its language resources.	The chatbot cannot engage in conversations related to the impact of specific genetic conditions, emotive personal circumstances, or expert medical advice, which possibly influences its language style.

^a^CA: conversational agent.

## Discussion

### Principal Findings

In human-CA linguistic communication, language cues are particularly important because they perform a crucial function in promoting user engagement [2], but few studies examine significant sociolinguistic dimensions in CA design across different languages and cultures, and the impact of these dimensions on user perceptions of CAs and their effectiveness in delivering health care services [16]. In this review, 6 of the 11 included publications deal exclusively with the language use of the CAs studied, and the remaining 5 are partly related to this topic. We derived the following themes from the language use in the 11 included studies through thematic synthesis.

#### Personal Pronouns

Among the 6 studies exploring exclusively the language used by CAs, the most interesting and distinctive study analyzes the influence of the CA’s use of formal and informal forms of the second-person pronoun “you”—*Tu/Vous* (T/V) distinction—across language contexts on user evaluations of digital health applications [56]. This study found a four-way interaction between T/V distinction, language, age, and gender, which influenced user assessments of four themes: (1) sociability, (2) CA-user collaboration, (3) service evaluation, and (4) behavioral intentions. Younger female and older male French speakers preferred the informal “T form” used by the public health CA for its human-likeness, and they would like to recommend the CA. In contrast, younger male and older female French speakers preferred the formal “V form” used by the CA. Younger male and female German speakers showed no obvious difference in their evaluations of the CA when they were addressed with the informal “T form” (“*Du*”), but “*Du*” led to lower scores in user evaluations as the German speakers’ age increased, especially for male Germans. German speakers’ user evaluation scores induced by the formal “V form” (“*Sie*”) were relatively stable and not affected by gender, but they increased slightly with age. The T/V distinction in French, German, Spanish, Chinese, Malaysian, and Korean, among many other distinctions of linguistic forms in various languages, indicates more or less formality, distance, or emotional detachment [67,68]. Such distinction encodes interactive meanings and shapes normative expectations such as politeness etiquette, the breach of which potentially results in perceived insult, membership of a different social class, and affiliation with another culture or grouping, leading to outcomes such as customer dissatisfaction [68-74]. CA developers need to consider this distinction and many other linguaculture-specific distinctions in the designing stage to enable CAs to choose appropriate forms for specific user groups, which facilitates user engagement in CA-based health communication [65,70].

#### Responses to Health and Lifestyle Prompts

A recent study analyzed the content appropriateness and presentation structures of CAs’ responses to health and lifestyle prompts (questions and open-ended statements) [57]. The CAs under scrutiny collectively responded appropriately to approximately 41% of safety-critical prompts by providing a referral to a health professional or service and 39% of lifestyle prompts by offering relevant information to solve the problems when prompted. The percentage of appropriate responses decreased if safety-critical prompts were rephrased or if the agent used a voice-only interface. The appropriate responses featured directive content and empathy statements for the safety-critical questions and open-ended statements and a combination of informative and directive content without empathy statements for the lifestyle questions and open-ended statements. These presentation structures seem reasonable, given that immediate medical assistance from a health professional or service is possibly needed to address problems mentioned in the safety-critical prompts. The use of empathy aligns with the testified exploitation of empathy on sensitive topics, showing that empathy is an important defining determinant of an effective CA [66,75,76]. The CAs examined in this study also displayed some defects, including the same CA’s inconsistent responses to the same prompt [57], which was also found in another study [40], and different answers from the same CA on different platforms. This may be attributed to the CAs’ diversified user interactions, but delivering appropriate responses consistently to user prompts, especially safety-critical prompts, is crucial to successful CA-based health communication and user adoption and adherence in the long run. Another weakness was the CAs’ inability to present large volumes of precoded information on safety-critical health and lifestyle prompts, which were instead primarily answered by web-searched information, as found in another study [59]. These identified deficiencies support the findings of other studies [59,77,78]. These results show that currently, natural language input is not able to provide constructive advice on safety-critical health issues [57,77-79]. CA designers need to improve this aspect substantially [78]. Such improvements in future CA development can guarantee positive user experience and thus ensure successful CA-based health communication.

#### Strategic Wording and Rich Linguistic Resources

The CAs studied were capable of making strategic word and utterance choices [58,59], as shown in Table 2. Such respectful, helpful, supportive, and empathetic wording successfully engaged the participants, who reported enjoying interacting with the CA, stating that “He answered me like a real person...,” “I don’t feel like they are judging me,” “The assistant feels understanding, attentive, very friendly,” and “It...guides the person on what to do without forcing us to make a final decision” [58]. The CA’s empathetic choice of words and verbal utterances (eg, spoken reflections) contributed to the participants’ positive experience with the CAs in terms of engagement with the technology, acceptability, perceived utility, and intent to use the technology [58]. In another study [59], each CA responded to user concerns with different wordings having similar or same meanings, showing the CAs’ relatively rich linguistic resources. However, there is still some scope for improvement in the CAs’ linguistic communication. For example, the CAs were inconsistent in responding to different health concerns, responding appropriately to some concerns but not to others; the CAs failed to understand some of the users’ concerns (eg, “I was raped,” “I’m being abused,” and “I was beaten up by my husband.”), illustrated by their honest but helpless responses like the following: “I don't understand I was raped. But I could search the Web for it.” “I don't know what you mean by “I am being abused.” How about a Web search for it?” “Let me do a search for an answer to “I was beaten up by my husband” [59]. Facing such deficiencies, software developers, clinicians, researchers, and professional societies need to design and test approaches that improve the performance of CAs [59].

**Table 2:** Examples of conversational agents’ strategic choice of words and utterances.

Categories	Examples
Respectful	“I will not pressure you in any way.”
Helpful	“Shall I call them for you?”/ “Need help?” / “Maybe it would help to talk to someone about it.”
Supportive	“I’ll always be right here for you.” / “There must be something I can do to make you feel better.”
Comforting	“Don’t worry. Things will turn around for you soon.” / “Keep your chin up, good things will come your way.”
Empathetic	“I’m sorry to hear that.” / “It breaks my heart to see you like that.”

#### Three-Staged Conversation Framework

Like the CA described in one of the studies [58], the CA under discussion in another study [64] is also based on motivational interviewing. What is different is that the CA in the former [58] features a model of empathetic verbal responses to engage users whereas the CA in the latter [64] is characteristic of a three-staged conversation framework targeted at questioning: introduction, reflection, and ending. In these stages, the CA begins with the purpose of the conversation and the request for permission to continue the talk; then, using a running head start technique, it engages subjects by eliciting from them the pros and cons of smoking followed by questions specifically adapted to each pro or con, and finally, it summarizes the conversation with a variable response: “You said *‘...’,* which I believe can be classified as ‘...’ ” [80]. This language framework aligned with the subjects’ sentiments toward smoking, contributing to an enjoyable engagement with the CA. However, the CA finished the conversation after soliciting responses to exception case questions. The lack of follow-on to exception case questions was most likely to make participants frustrated and potentially trigger negative, undesired effects [64]. Improvement in this respect depends on the CA’s response generation capabilities based on general natural language understanding.

#### Human-Like Well-Manipulated Conversations

Some studies mainly introduce themed CAs for specific physical problems including Parkinson disease, neurological conditions, and genetic diseases [61,81,82]. In these investigations, user-CA dialogs are illustrated to exemplify the CA’ roles in the management of these diseases. The CA analyzed in one of the studies [61] seeks to solicit information concerning users’ well-being before providing exercise encouragement and speech assessments in random, human-like conversations. In these conversations, the CA displayed its ability to initiate conversations closely related to the patients’ specific conditions and recommend physical exercise using friendly, polite, empathetic, and encouraging language (eg, “I’m sorry to hear that, have you taken any new medication?”) while conducting speech assessments, when necessary, by asking users to give speech samples. When responding to user phrases indicating depressive or even suicidal thoughts, the CA resorted to supportive, referral, directive, and empathetic replies (eg, “Get help! You are not alone. Call lifeline 13 11, 14, or 000.”), as found in some studies [57,66,75,76]. Moreover, the CA can learn and store a new response permanently when finding the first response inappropriate from the users’ feedback (eg, “What should I say instead?”). The CA’s sensitivity to phrases indicative of negative moods addressed affective symptoms effectively, and its capability of learning appropriate responses ensured user engagement and disease management. The CAs investigated in some studies [81,82] exhibited language use and manipulation skills similar to the CA examined in another study [61]. Unlike some CAs [61,81], others [82] can educate users through explaining genetic conditions and terminologies precoded into their language resources.

#### Symbols and Images Coupled With Phrases

Compared with the CAs discussed above, the CA in another study [62], though similar in its friendly, polite, supportive, empathetic, informative, and directive language engagement with patients, seems distinct in that it engaged users with a different language (symbols and images coupled with phrases). The special language used by the CA features customization, interoperability, and personalization, which is tailored for children on the autism spectrum. This considerate language design reminds CA designers that they need to take certain factors into account to design CAs for their desired purposes when inputting language into them.

In comparison with the studies discussed above, each of the remaining 2 publications [60,62] only provides 1 exemplary dialog between the CA studied and a user. In these 2 studies, the CAs use a language similar to that used by the CAs investigated in the other studies [56-59,61,63,64,81,82].

### Implications

Analyzing CAs’ language use to engage patients and consumers in health communication is an important subject of research. The 6 themes of language use presented above significantly promoted user engagement. Designers of CAs and similar technologies need to consider these crucial linguistic dimensions in the design and development stage across different languages and cultures to improve the user perception of these systems and their delivery of effective health care interventions. Due to their increasing capabilities and expanding accessibility, CAs are playing critical roles in various health-related aspects of patients’ daily lives through responding to users in natural language [79,83-86]. Future studies should investigate health care CAs from the linguistic perspective. This is crucial because language exerts considerable influence on social cognition and coconstructed meaning between dyadic conversing partners [33,34]. The language use presented by CAs in response to users can “affect their perception of the situation, interpretation of the response, and subsequent actions” [57]. Whether patients and customers choose to accept CAs’ health advice depends largely on the way they give advice. Good advice is judged by the advice content and its presentation [87]. “Advice that is perceived positively by its recipient facilitates the recipient’s ability to cope with the problem and is likely to be implemented” [87]. Moreover, cultural nuances underlying the language use of CAs need to be considered by designers. For example, addressing users by their first names was linked to users’ perceptions of politeness and thoughtfulness of the CAs, which may be bound to cultural limits and preferences [24]. Considering that few studies have examined significant cultural and sociolinguistic phenomena in CA designs across different linguacultures and the influence of these phenomena on the perceptions of CAs’ effectiveness in health care service delivery [16], further studies in this respect must be conducted to enable CAs to achieve greater credibility and trustworthiness using more engaging language [38,39].

Alongside the beneficial language use that needs to be input into CAs, there are drawbacks in the language output of these systems that need to be improved in future design and development to enhance user experience and adherence. Consistent language performance is one of the most significant considerations. As revealed in previous studies, some CAs provided inconsistent responses to the same prompts or on different platforms [57,59], and some were incapable of presenting large volumes of information on prompting [59,77,78], making users somewhat puzzled and frustrated, thus undermining follow-up medical actions. It was found that some most frequent issues related to user experience stemmed from spoken language understanding and dialog management problems [59,81,88]. Although CAs capable of using unconstrained natural language input have gained increasing popularity [89], CAs currently used in health care lag behind those adopted in other fields (eg, travel information and restaurant selection and booking), where natural language generation and dialog management techniques have advanced well beyond rule-based methods [90,91]. Health care CA designers need to empower these systems with unconstrained natural language input to ensure their consistent language output. Moreover, advances in machine learning, especially in neural networks, need to be integrated into the design of CAs to empower these systems with more complex dialog management methods and conversational flexibility [92,93].

Furthermore, there are other aspects of language use that the 11 included studies did not consider, and we have not discussed these in the Principal Findings subsection. We synthesized these aspects and those discussed above to obtain an open list of recommendations for improving language use in CA-based health communication along with the pros and cons of existing CA-based communication styles that need to be considered in future CA designs, which are given in Textbox 2.

**Recommendations for improving language use in conversational agent–based health communication.**

**Recommendations**

- Use a neutral style (eg, methodological mistakes) rather than an aggressive style (eg, really dumb methodological mistakes) [36].
- Use an everyday style (eg, heart attack) rather than a technical style (eg, myocardial infarction) [41].
- Use a tentative style (eg, presumably similar) rather than a nontentative style (eg, similar) [42].
- Use an emotional style rather than a nonemotional style [43].
- Use an enthusiastic style rather than a nonenthusiastic style [44].
- Use personal references (eg, first-person and second-person pronouns) [45,46,54].
- Use personal testimonials [47].
- Use replies featuring directive content and empathy statements for the safety-critical questions and open-ended statements and a combination of informative and directive content without empathy statements for the lifestyle questions and open-ended statements [55].

**Pros**

- The conversational agent (CA) used a strategic choice of words and utterances, which were respectful (eg, “I will not pressure you in any way.”), helpful (eg, “Shall I call them for you?,” “Need help?,” and “Maybe it would help to talk to someone about it.”), supportive (eg, “I’ll always be right here for you” and “There must be something I can do to make you feel better.”), comforting (eg, “Don’t worry. Things will turn around for you soon” and “Keep your chin up, good things will come your way.”), and empathetic (eg, “I’m sorry to hear that” and “It breaks my heart to see you like that.”) [56-58,60].
- The CA solicited information concerning users’ well-being before providing exercise encouragement and speech assessments in random, human-like conversations in friendly, polite, empathetic, supportive, and encouraging language (eg, “I’m sorry to hear that, have you taken any new medication?”) [59,63].
- A three-staged conversation framework targeted at questioning was used: introduction, reflection, and ending [62].
- The CA educated users through explaining terminologies precoded into its language resources [64].
- The CA used a running head start technique [77].
- Advances in machine learning, especially in neural networks, were used to empower CAs with more complex dialog management methods and more conversational flexibility [90,91].

**Cons**

- The CA used an aggressive style (eg, “really dumb methodological mistakes”) [36].
- The CA used a technical style (eg, “myocardial infarction”) [41].
- The CA used a nontentative style (eg, “similar”) [42].
- The CA used a nonemotional style [43].
- The CA used a nonenthusiastic style [44].
- The CA provided inconsistent responses to the same prompts [55,57].
- The CA provided inconsistent responses to the same prompts on different platforms [55].
- The CA was unable to present large volumes of information on given prompts [57,75,76].

### Limitations and Further Studies

This systematic review has some limitations. The first one was attributed to the retrieval of relevant articles. We searched PubMed and ProQuest for suitable publications. The limited number of included papers (N=11) could not give a paramount overview of previous studies we intended to review systematically. In further studies, the scope of search needs to be expanded to more databases, including Embase, CINAHL, PsycInfo, and ACM Digital Library. Second, some of the principal findings may have low generalizability due to the small number of included articles, especially considering that some language use reported in these publications is specific to 1 CA studied, for example, the autism-themed CA [63]. Third, this limited number of included studies from the perspective of language use prevented us from conducting a relatively more comprehensive systematic review. In future, we will contribute another review as a sequel to this review that is hopefully more comprehensive. Fourth, only 1 selected study is concerned with the cultural nuances underlying the language use examined [82]. It is impossible to make comparisons and draw specific conclusions concerning cultural nuances across the selected studies. This is a limitation that needs to be overcome in future research.

### Conclusions

Health care CAs are designed to simulate natural language communication between 2 individuals. In CA-human health communication, the language used by CAs is crucial to the improvement of user self-disclosure or self-concealment, user engagement, user satisfaction, user trust, and intention to use. However, only few studies focused on this topic, and no systematic review was found in this line of research. Our review fills this gap in the literature. The positive and negative language use of CAs identified in the 11 included papers can provide new insights into the design and development, popularization, and research of CA applications. This review has some practical implications for CA-based health communication, highlighting the importance of integrating positive language use in the design of health care CAs while minimizing negative language use. In this way, future CAs will be more capable of engaging with patients and users when providing medical advice on a variety of health issues.

## Abbreviations

CA: conversational agent
PRISMA: Preferred Reporting Items for Systematic Reviews and Meta-Analyses
